# MiR-26a Inhibits Proliferation and Migration of Breast Cancer through Repression of MCL-1

**DOI:** 10.1371/journal.pone.0065138

**Published:** 2013-06-04

**Authors:** Jie Gao, Laisheng Li, Minqing Wu, Min Liu, Xinhua Xie, Jiaoli Guo, Hailin Tang, Xiaoming Xie

**Affiliations:** 1 Department of Breast Oncology, Sun Yat-sen University Cancer Center, Guangzhou, People’s Republic of China; 2 Department of Laboratory Medicine, The First Affiliated Hospital of Sun Yat-sen University, Guangzhou, People’s Republic of China; 3 State Key Laboratory of Oncology in South China, Sun Yat-sen University Cancer Center, Guangzhou, People’s Republic of China; H. Lee Moffitt Cancer Center & Research Institute, United States of America

## Abstract

Breast cancer is the most commonly malignancies in women. MicroRNAs are a family of small non-coding RNAs 18–25 nucleotides in length that post-transcriptionally modulate gene expression. MiR-26a has been reported as a tumor suppressor microRNA in breast cancer, which is attributed mainly to targeting of MTDH and EZH2, however, the expression profile and therapeutic potential of miR-26a is still unclear. Here we demonstrate that miR-26a is down-regulated in breast cancer cells and clinical specimens and its modulation in breast cancer cells regulates cell proliferation, colony formation, migration and apoptosis. MCL-1, an anti-apoptotic member of the Bcl-2 family, as novel targets of miR-26a was found to be in reverse correlation with ectopic expression of miR-26a and knockdown of MCL-1 phenocopied the effect of miR-26a in breast cancer cell lines. It was further explored that miR-26a increased sensitivity of breast cancer cells to paclitaxel in which MCL-1 was involved. Thus, miR-26a impacts on cell proliferation and migration of breast cancer by regulating several carcinogenesis-related processes, including a novel mechanism involving the targeting of MCL-1.

## Introduction

Breast cancer is the most commonly diagnosed cancer in women and the second leading cause of cancer deaths in the developed world. [1] Although advances in both diagnosis and comprehensive treatment, which incorporates surgery, radiation therapy and chemotherapy, lead to the improvement of prognosis, [2], [3] chemotherapy resistance and metastasis remain to be major challenges in breast cancer therapy. [2], [3], [4], [5] Once recurrence or distant relapse occurs due to chemotherapy-resistant or metastatic cells, conventional therapy is nearly ineffective. [5] Therefore, the development of novel effective therapeutic strategies is essential and urgent.

MicroRNAs (miRNA) represent a diverse class of endogenous noncoding RNAs 18–25 nucleotides in length which post-transcriptionally regulate gene expression. [6] Evidence has shown that each miRNA regulates hundreds of target genes [6], [7] and 52.5% of miRNAs are located in cancer-associated genomic regions. [8] Therefore, a large amount of miRNAs are involved in tumorigenesis and function as oncogenes or tumor suppressor genes depending on their targets. [9] Researchers have identified dysregulation of several miRNAs in breast cancer, such as miR-21 [10], miR-34a [11], [12], miR-101 [13], miR-122 [14] and miR-155 [15], which contribute to the progression of malignancy. A recent study has suggested miR-26a is downregulated in breast cancer and functions as a tumor suppressor [16], however, the expression profile and therapeutic potential of miR-26a remains unclear.

The global downregulation of miRNAs [17] including miR-26a draws the attention to high-expression genes computationally predicted as putative miR-26a targets in human breast cancer. Apart from MTDH [16] and EZH2 [18], MCL-1(myeloid cell leukemia 1), a pro-survival member of the Bcl-2(B-cell CLL/lymphoma 2) family, is expected to be highlighted due to the association between the aberrant expression of pro-survival Bcl-2 family proteins and tumorigenesis and resistance to chemotherapeutics [19]. And it has been demonstrated that several miRNAs induces apoptosis by targeting MCL-1 in gastric cancer [20], hepatocellular carcinoma [21], nasopharyngeal carcinoma [22], and multiple myeloma [23].

In this study, we investigated the expression level of miR-26a in breast cancer cells and tissues as well as its effect on cell proliferation, colony formation, migration and apoptosis. Further, we explored the underlying mechanism of miR-26a functions in breast cancer. In addition, we examined the synergistic anti-tumor effects of miR-26a with paclitaxel in breast cancer cells. Our findings will provide a clear understanding of breast cancer pathogenesis as well as suggest that miR-26a may act as a suppressor which can be a novel potential target for breast cancer therapy.

## Materials and Methods

### Clinical Specimens

Breast cancer biopsy specimens and normal biopsies of the breast were obtained from Sun Yat-sen University Cancer Center (Guangzhou, China), fixed in RNAlater (Ambion, Austin, TX, USA) immediately after biopsy and stored at −80°C until use. Both tumor and normal tissues were histologically confirmed by two different experienced pathologists according to the World Health Organization (WHO) with H&E (hematoxylin and eosin) staining. Informed consent was obtained from each patient, and the research was approved by the Research Ethics Committee of Sun Yat-Sen University Cancer Center.

### Immunohistochemical Staining

Immunohistochemical staining (IHC) was performed as previously reported [11]. Briefly, paraffin-embedded tissues were sectioned at 5 µm, deparaffinized, rehydrated through graded alcohols and subjected to antigen retrieval in heated citrate buffer. Following a blocking step, the slides were incubated with primary antibody, washed, biotinylated secondary antibody was applied and the immunocomplexes were visualized using an avidin-biotin complex immunoperoxidase system (Vector Laboratories, Burlingame, CA, USA) with 0.03% diaminobenzidine (DAB) as a chromagen and hematoxylin as the counterstain. Both external and internal controls were used to assess the quality of the IHC reaction. For evaluation of the staining the whole area of the section was used. If more than 10% of the cancer cells contained immunostaining, the patient was classified as MCL-1 positive; if less than 10% of the cancer cells contained immunostaining, the patient was classified MCL-1 negative. Two pathologists who were blinded to all clinical information scored all specimens. Conflicts (about 5% of cases) were resolved by consensus.

### Cell Culture and miRNA Transfection

The human breast cancer cell lines MDA-MB-231, MCF-7, MDA-MB-435, MDA-MB-468 and two immortalized normal mammary epithelial cell lines MCF-10A and 184A1 were all obtained from American Type Culture Collection, and freshly recovered from liquid nitrogen (<3 month). The breast cancer cells were cultured in Dulbecco’s modified Eagle’s medium (DMED, Invitrogen, CA, USA) supplemented with 10% fetal bovine serum (FBS, GIBCO, Cappinas, Brazil). MCF-10A cells were cultured in Keratinocyte-SFM (Invitrogen, CA, USA) supplemented with pre-qualified human recombinant epidermal growth factor 1–53 (EGF 1–53, Invitrogen, CA, USA) and bovine pituitary extract (BPE, Invitrogen, CA, USA). 184A1 cells were cultured in Mammary Epithelium Basal Medium (MEBM, Clonetics, MD, USA). All cells were cultured in a humidified incubator at 37°C containing 5% CO_2_.

The miR-26a mimics and a non-specific miR control (miR-Ctrl) were synthesized and purified by RiboBio (Guangzhou, China). MiRNA mimics were transfected at working concentrations using Lipofectamine 2000 reagent (Invitrogen, CA, USA).

### MTT Assay

Cells were plated in 96-well plates at 5×10^3^ per well in a final volume of 100 µL and treated with miRNAs or paclitaxel. After incubation for 24, 48, 72 and 96 hours in a humidified incubator at 37°C with 5% CO_2_, 3-(4,5-dimethylthiazolyl-2-yl)-2-5 diphenyl tetrazolium bromide (MTT, Sigma, St. Louis, MO, USA) was added. The plates were incubated for another 4 hours, replaced with dimethyl sulfoxide (DMSO, Sigma, St. Louis, MO, USA) after removal of culture medium, and the absorbance was measured at 570 nm by SpectraMax M5 Microplate Reader (Molecular Deviced, Sunnyvale, CA, USA).

### Colony Formation Assay

Cells were seeded in 6-well plates at 2×10^2^ per well 72 hours after transfection, incubated for 2 weeks. Then the cells were washed twice with PBS, fixed with methanol/acetic acid (3∶1, v/v), and stained with 0.5% crystal violet. The number of colonies was counted under the microscope (Olympus IX81, Tokyo, Japan).

### Apoptosis Assay

Annexin V/propidium iodide (PI) staining was performed for the detection of apoptotic cells. After 48 hours of transfection, 5×105 cells were collected and washed twice with ice-cold PBS. The cells were then stained using the Alexa Fluor®488 annexin V/Dead Cell Apoptosis Kit with Alexa® Fluor 488 annexin V and PI for Flow Cytometry (Invitrogen, CA, USA) according to the manufacturer’s guidelines. The untreated cells served as a negative control for the double staining. Flow cytometric analysis was performed immediately using a FACSCalibur instrument (Becton Dickinson, CA, USA).

### Wound Healing Assay

MiRNA-transfected cells were scratched using a standard 200 µL tip. The debris was removed by washing with serum-free medium. Serial photographs were obtained at different time point using a phase contrast microscope (Olympus IX81, Tokyo, Japan).

### RNA Isolation, Reverse Transcription, and Quantitative Real-time PCR

Total RNA was extracted using Trizol Reagent (Invitrogen, CA, USA). To quantitate miR-26a expression, reverse transcription was performed with a specific stem-loop real-time PCR miRNA kit (RiboBio, Guangzhou, China). Quantitative real-time PCR (qPCR) was performed using Platinum SYBR Green qPCR SuperMix-UDG system (Invitrogen, CA, USA) on an ABI7900HT System. 5S rRNA was used as an endogenous control. All samples were normalized to internal controls and fold changes were calculated through relative quantification (RQ = 2^−ΔΔCT^).

### Western Blot Analysis

Protein lysates were separated by 10% SDS-PAGE and electrophoretically transferred to polyvinylidene difluoride membrane (PVDF, Millipore, MA, USA). Then, the membrane was incubated with rabbit polyclonal antibody against human MCL-1 (Santa Cruz, CA, USA) followed by horseradish peroxidase (HRP)-labeled goat-antirabbit IgG (Santa Cruz, CA, USA) and detected by chemiluminescence. Glyceraldehyde-3-phosphate dehydrogenase (GAPDH) was used as a protein-loading control. The specific protein bands were visualized using the Supersignal West Pico ECL chemiluminescence detection kit (Pierce, IL, USA) and Kodak X-ray film (Eastman Kodak Co, NY, USA).

### Statistical Analysis

SPSS 13.0 software was used for stastistical analysis. Data were presented as mean of 3 independent experiments. Two-tailed Student’s *t* test was used for comparisons of 2 independent groups. *P* values of <0.05 were considered statistically significant.

## Results

### MiR-26a was Down-regulated in Human Breast Cancer Cell Lines and Clinical Specimens

**Figure 1:**
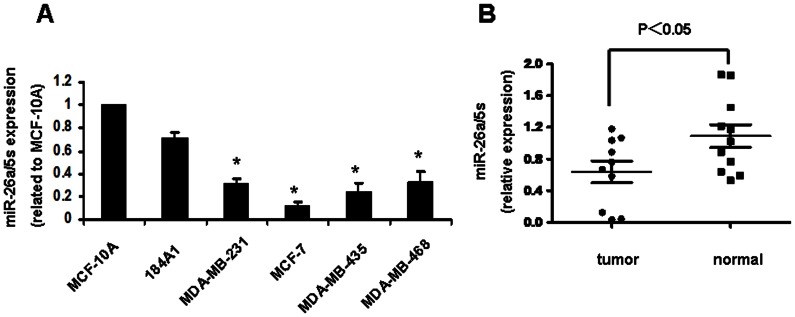
The expression of miR-26a was reduced in breast cancer cell lines and clinical specimens. A. Expression of miR-26a in the 2 immortalized normal mammary epithelium cell lines and 4 breast cancer cell lines. B. Average expression level of miR-26a in human breast cancer tissues and normal breast tissues. MiRNA abundance was normalized to 5s rRNA. *P<0.05 compared with control.

The expression of mi-26a was first evaluated in 4 breast cancer cell lines: MDA-MB-231, MCF-7, MDA-MB-435, MDA-MB-468, compared to that of 2 immortal nontumorigenic cell lines MCF-10A and 184A1. The result showed that the expression level of miR-26a was decreased significantly in all 4 breast cancer cell lines (Fig. 1A).

We further examined the expression level of miR-26a in 52 breast cancer specimens and 29 normal breast tissues. Similar to the data obtained from the cell lines, the average expression level of miR-26a was significantly lower in breast cancer specimens than in normal breast tissues (Fig. 1B; P<0.05). In addition, the tumor size of breast cancer patients, HER2 and Ki-67 status are significantly associated with the expression level of miR-26a (Table 1; P<0.05).

**Table 1:** The relationship between miR-26a expression and clinicopathological parameters in breast cancer.

Clinicopathologic parameters	Number of cases	Median expression of miR-26a	*P*-value
Age (years)			
≤45	31	0.4797±0.3127	0.135
>45	21	0.5733±0.2049	
Tumor Size (cm)			
≤2	25	0.5608±0.3276	0.036
>2	27	0.4774±0.2162	
TNM stage			
I+II	30	0.5303±0.2975	0.409
III+IV	22	0.5000±0.2492	
Lymph node metastasis			
No	27	0.5237±0.3162	0.481
Yes	25	0.5108±0.2310	
ER status			
Negative	22	0.5032±0.2991	0.810
Positive	30	0.5280±0.2623	
PR status			
Negative	28	0.5079±0.2882	0.858
Positive	24	0.5288±0.2665	
Her2 status			
Negative	34	0.5491±0.3134	0.014
Positive	18	0.4578±0.1785	
Ki-67 status			
≤20%	29	0.5607±0.3197	0.039
>20%	23	0.4630±0.2020	
MCL-1 status			
Negative	29	0.5734±0.3224	0.005
Positive	23	0.4470±0.1865	

### MiR-26a Reduced Viability and Clonogenicity of Breast Cancer Cells

**Figure 2:**
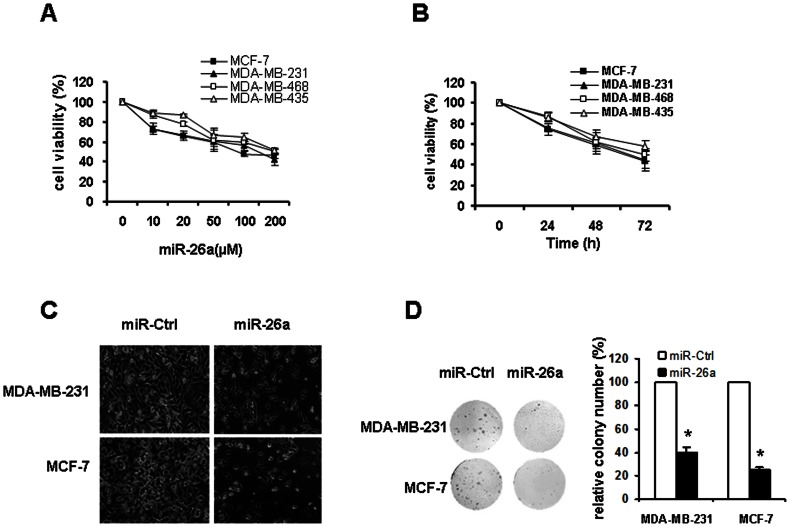
Overexpression of miR-26a lead to reduced cell viability and decreased clonogenicity. A. Dose effect. Cells were transfected with miR-26a at the indicated concentrations for 48 hours. B. Time effect. Cells were transfected with 50 µM of miR-26a for indicated periods. C. Morphologic changes of MDA-MB-231 and MCF-7 cells in response to miR-26a inhibition. D. Influence of miR-26a on colony formation of MDA-MB-231 and MCF-7 cells. Representative dishes are presented (left). The number of colony was counted for each well of six-well plates and the evaluation of colony numbers was shown in the y-axis of the right panel. *P<0.05 compared with control.

To explore the effect of miR-26a on cell growth, MDA-MB-231, MCF-7, MDA-MB-435 and MDA-MB-468 cells were transfected with miR-26a mimic. The impact of different doses of miR-26a was evaluated firstly. Cells were transfected with 0–200 µM miR-26a or miR-Ctrl for 48 hours and the relative viability was determined by MTT assay. As shown in Fig. 2A, miR-26a inhibited cell growth in a dose-dependent manner, compared with miR-Ctrl at any of the corresponding concentrations. Next, cells were transfected with 50 µM of miR-26a or miR-Ctrl for varying periods. The result of MTT assay indicated that miR-26a led to growth inhibition in all 4 breast cancer cell lines as early as 48 hours posttransfection, persisting for 72 hours (Fig. 2B).

In addition, the images of MDA-MB-231 and MCF-7 cells demonstrated that miR-26a-transfected cells displayed significantly reduced cell counts, which was concordant with the MTT data. Cells treated with miR-26a exhibited a morphological change with the extension of cytoplasmic portion and rounding of the cell body, while the rest of the spindled cells showed conspicuous shrinkage and extensive detachment (Fig. 2C).

We further performed colony formation assay to determine the effect of miR-26a on clonogenicity of breast cancer cells. As shown in Fig. 2D, miR-26a-transfected MDA-MB-231 and MCF-7 cells exhibited much fewer and smaller colonies compared with miR-Ctrl-transfected cells (P<0.05).

### MiR-26a Induced Significant Inhibition of Migration in Breast Cancer Cells

**Figure 3:**
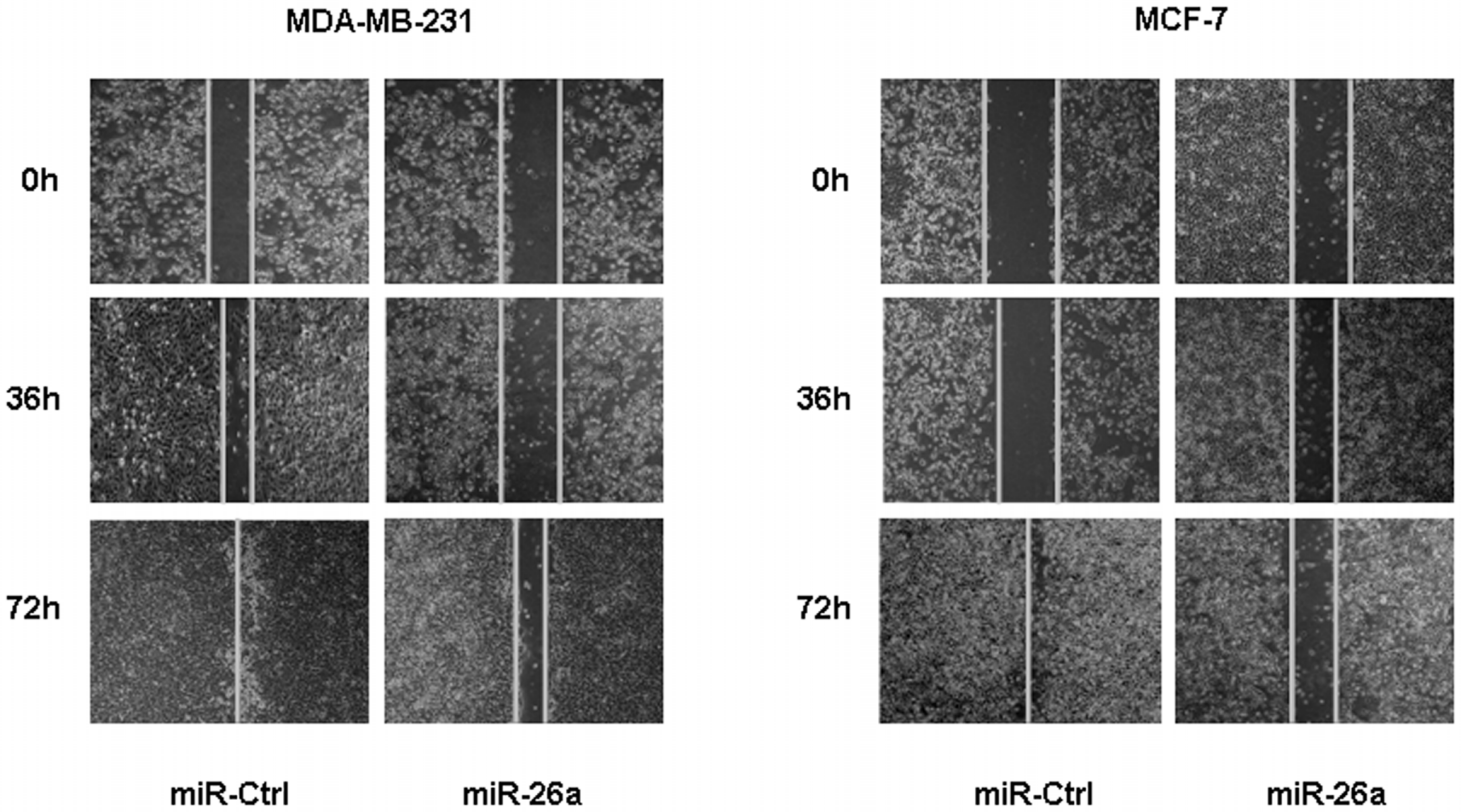
Ectopic restoration of miR-26a inhibited cell migration. In the wound healing assay, uniform scratches were made in MDA-MB-231 and MCF-7 cells, then serial photographs were obtained at indicated time posttransfection.

Given that cell migration is among the common functions required by tumor cells for metastatic progression [24], as well as the strong association between tumor cell migration and tumor cell invasion and metastasis [25], we asked whether miR-26a could affect breast cancer cell migration *in vitro*. In wound healing assay, ectopic restoration of miR-26a did obviously delay the closure rate of both MDA-MB-231 and MCF-7 cells forward the wound area, compared with cells transfected with miR-Ctrl (Fig. 3), which suggested that miR-26a has the ability to inhibit migration for breast cancer cells.

### MiR-26a Affected Apoptosis of Breast Cancer Cells *in vitro*

**Figure 4:**
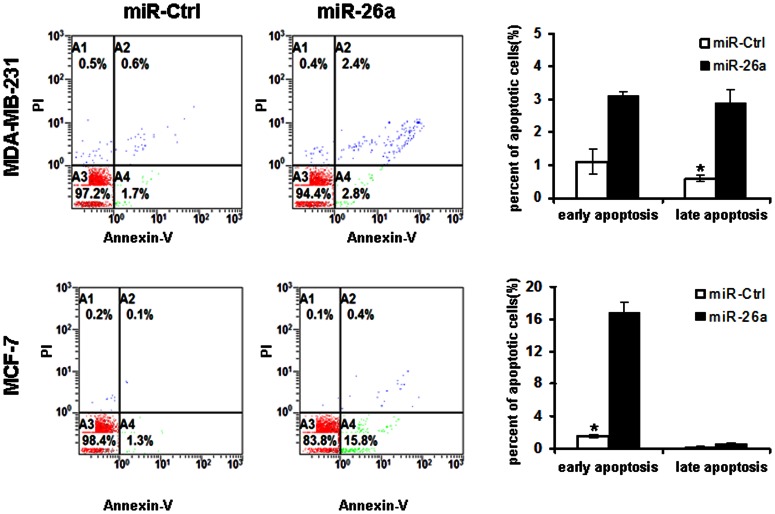
Effect of miR-26a on apoptosis in breast cancer cells. FACS analysis of MDA-MB-231 and MCF-7 cells transfected with miR-26a for 48 hours. The percentage of Annexin V-FITC positive cells to the total cells was shown in the bar graphs. *P<0.05 compared with control.

In order to further investigate the biological effect of miR-26a ectopic restoration on apoptosis in breast cancer cells, we transiently transfected MDA-MB-231 and MCF-7 cells with miR-26a and measured the apoptotic cell death assay using flow cytometric analysis of Annexin V-FITC/PI staining (Fig. 4). The cells in the lower right quadrant of the square chart represent the percentage of early apoptotic cells and in the upper right quadrant represent late apoptotic cells.

Compared to miR-Ctrl, transfection with miR-26a obviously increased the number of early apoptotic cells in MCF-7 (1.5% vs 16.8%; P<0.05) and late apoptotic cells in MDA-MB-231 (0.6% vs 2.9%; P<0.05). There was no significant difference of the number of early apoptotic cells in MDA-MB-231 and late apoptotic cells in MCF-7. These results showed that up-regulation of miR-26a can promote breast cancer cell apoptosis.

### MiR-26a Regulated Endogenous MCL-1 Expression in Breast Cancer Cells *in vitro*

**Figure 5:**
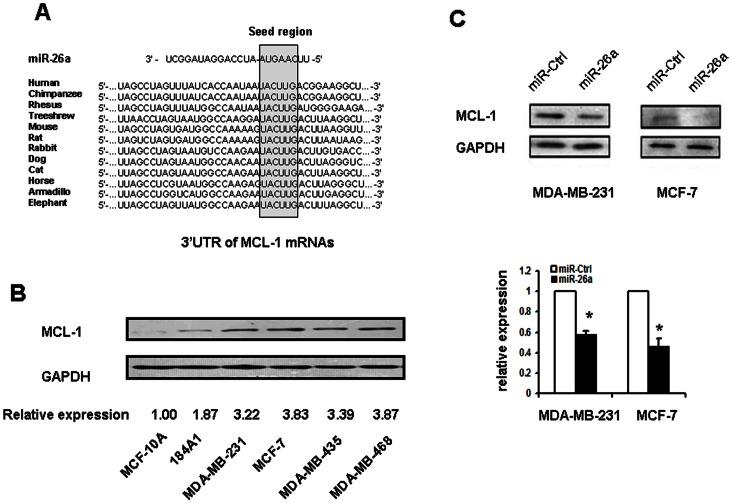
MCL-1 is the target of miR-26a. A. Putative miR-26a binding sites in the 3′UTR region of MCL-1 and interspecies conservation of seed matching sequences (gray box). B. Expression of MCL-1 in the 2 immortalized normal mammary epithelium cell lines and 4 breast cancer cell lines. C. Western blot assay for MCL-1 expression after MDA-MB-231 and MCF-7 cells were transfected with miR-26a for 48 hours. *P<0.05 compared with control.

To figure out the mechanism by which miR-26a may perform in breast cancer, we made an online search of miR-26a targets by Targetscan. Among the hundreds of putative miRNA targets, we focused on MCL-1 as it is an anti-apoptotic member of the Bcl-2 family, which has been validated to be linked with tumorigenesis and resistance to chemotherapy by large amounts of evidences. Target scan showed that there was a miR-26a responsive sequence in 3′-UTR (3′untranslated region) of MCL-1, which was highly conserved among different species (Fig. 5A).

In order to determine whether low expression level of miR-26a is associated with high expression level of MCL-1 in breast cancer cells, we further performed Western blot analyses in the 2 immortalized normal mammary epithelium cell lines and 4 breast cancer cell lines. The result showed that the endogenous MCL-1 expression was obviously increased in all 4 breast cancer cell lines (Fig. 5B). In addition, IHC was carried out to detect MCL-1 expression in breast cancer tissues which were used for miR-26a detected. Compared with the MCL-1 negative group patients, the tumors of the MCL-1 positive group patients had a lower miR-26a expression level (Table 1, P<0.01).

Then we transfected MDA-MB-231 and MCF-7 cells with miR-26a or miR-Ctrl and examined MCL-1 expression level by Western blot analyses. As shown in Fig. 5C, the level of MCL-1 protein was significantly down-regulated by transient transfection of miR-26a in both two cell lines (P<0.05), which means the expression of endogenous MCL-1 was regulated by miR-26a in breast cancer cells.

Knockdown of MCL-1 suppressed cell growth, proliferation and induced cell apoptosis.

**Figure 6:**
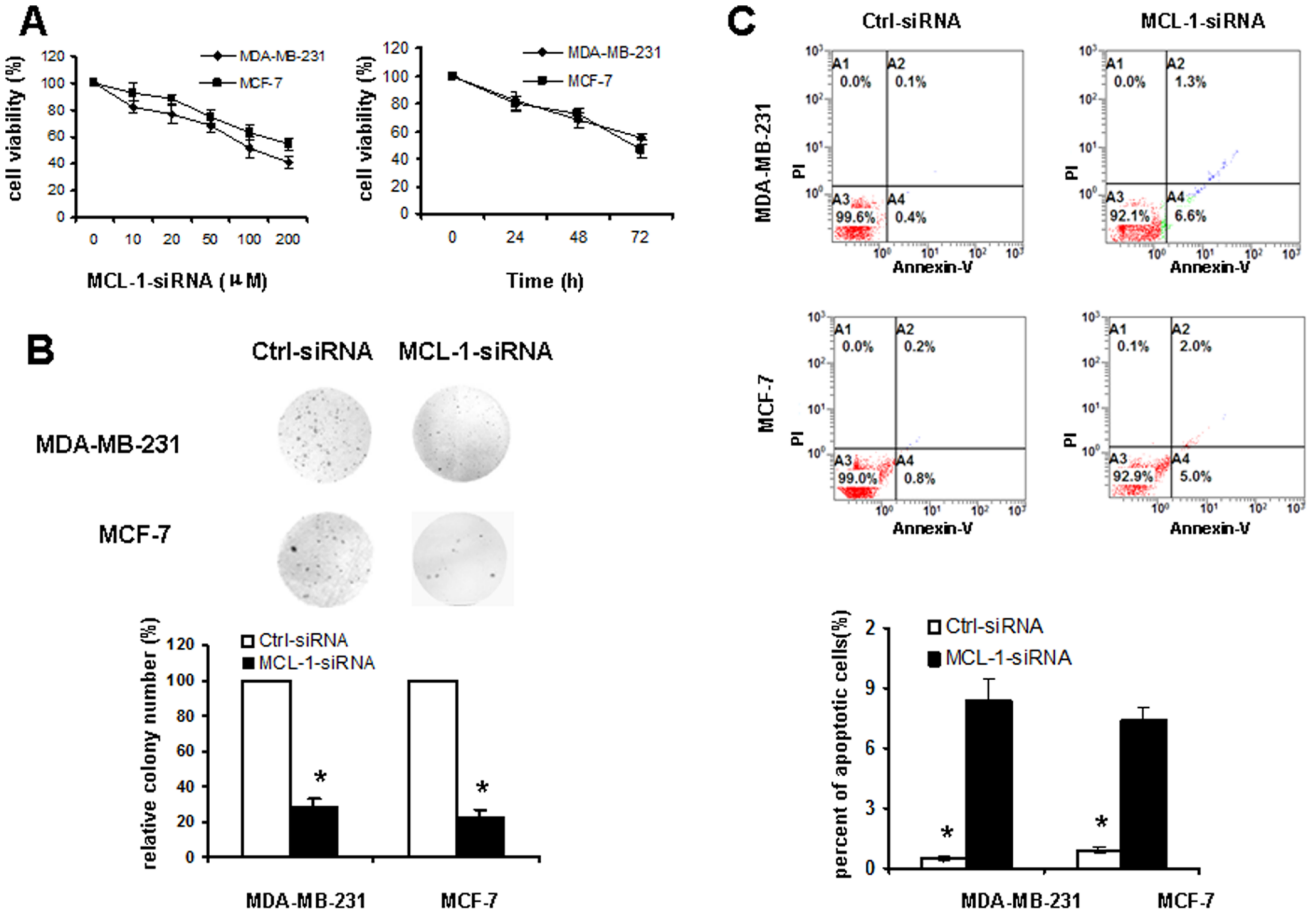
Knockdown of MCL-1 suppresses cell proliferation, clonogenicity and induces cell apoptosis. A. Does effect and time effect of transfection of MCL-1-siRNA on the proliferation of MDA-MB-231 and MCF-7 cells. B. The functional role of MCL-1 in breast cancer cell growth was analyzed by colony formation of MDA-MB-231 and MCF-7 cells. The evaluation of colony numbers was shown in the panel. C. Influence of MCL-1 on apoptosis in breast cancer cells was monitored by flow cytometry. The percentage of Annexin V-FITC positive cells to the total cells was shown in the bar graphs. *P<0.05 compared with control.

In order to address the functional role of MCL-1 in breast cancer cells, MDA-MB-231 and MCF-7 cells were transfected with MCL-1-specific siRNAs. The results of MTT assay indicated that knockdown of MCL-1 led to inhibition of cell growth and proliferation both in these two cell lines (Fig. 6A). A colony-forming assay was carried out to evaluate the effect of MCL-1 on the clonogenicity ability of breast cancer cells. As shown in Fig. 6B, MCL-1-siRNA-transfected cells displayed fewer and smaller colonies compared with control-siRNA transfectants (P<0.05). Furthermore, flow cytometry analysis revealed that transfection with MCL-1-siRNA significantly increased the number of apoptotic cells in both MDA-MB-231 (0.5% vs 8.4%; P<0.05) and MCF-7 (0.9% vs 7.4%; P<0.05) cells(Fig. 6C).

### MiR-26a Increased Sensitivity to Paclitaxel in Breast Cancer Cells

**Figure 7:**
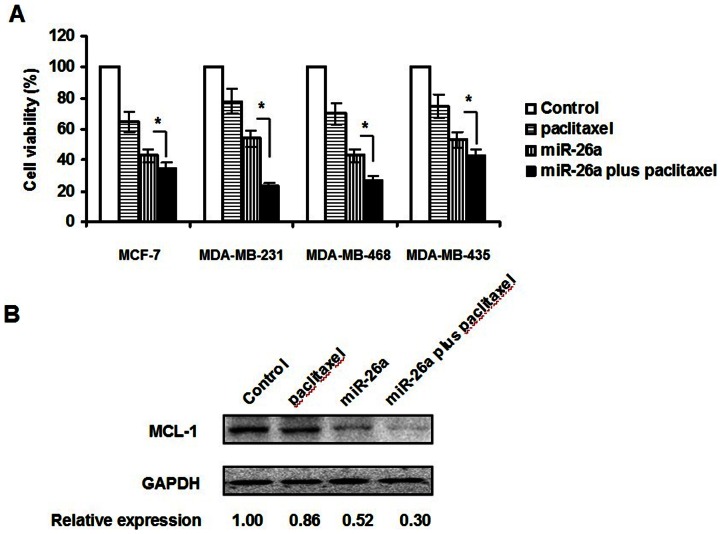
MiR-26a sensitized breast cancer cells to paclitaxel. A. Viability of MDA-MB-231, MCF-7, MDA-MB-435, MDA-MB-468 cells was determined by MTT assay 72 hours after treatment. The percentage of the cell viability as compared with itself without paclitaxel treatment was presented. The concentrations of miR-26a and paclitaxel were 50 µM and 0.12 nM, respectively. B. Western blot assay for MCL-1 expression 48 hours after treatment in MDA-MB-231 cells. *P<0.05.

Given that paclitaxel was involved in the majority of strategies of conventional chemotherapy, and several miRNAs have been reported to have the synergistic anti-tumor effects with conventional chemotherapy [26], the raised question was whether miR-26a could enhance the therapeutic effect of paclitaxel in breast cancer cells. In order to find the answer, MTT assay was performed 72 hours after the treatment of miR-26a and paclitaxel. As shown in Fig. 7A, the combination treatment caused greater inhibition of cell growth, as compared with paclitaxel alone. This result suggested that miR-26a effectively sensitized breast cancer cells to paclitaxel.

To further determine whether MCL-1 was involved in this function of miR-26a, we examined MCL-1 expression level by Western blot analyses in MDA-MB-231 cells. As shown in Fig. 7B, the level of MCL-1 protein was significantly down-regulated by the combination treatment of miR-26a and paclitaxel, as compared with paclitaxel alone.

## Discussion

Breast cancer is the most frequent cancer of women, which covers 23% of all cancers [27]. It is a heterogeneous disease due to complicated etiology involving both genetic and environmental factors. Despite the advances of both diagnosis and treatment, breast cancer remains incurable. MiRNAs are a class of multifunctional small molecules that regulate the stability or translational efficiency of targeted messenger RNAs. Mature miRNAs function in the form of RNA-induced silencing complex (RISC) [28]. The miR-RISC leads to base-pairing interactions between microRNAs and the 3′UTR of the target mRNA, which often repress the gene translation or cleave the target mRNA. [29] It is predicted that miRNAs play pivotal roles in regulating wide biological processes, ranging from cell proliferation, apoptosis, cell cycle progression to cancer initiation and metastasis [30], [31]. Moreover, the prominent functions of miRNAs in cancer diagnosis, pathogenesis and prognosis have been demonstrated. [12], [32], [33].

Since the first time was breast cancer reported to be linked with the altered expression of miRNA in 2005 [34], increasing miRNAs were proved to have close relationship with breast cancer. Unlike several most intensively studied miRNAs, such as let-7 and miR-34a, both of which were demonstrated to be tumor suppressor and decreased in majority cancers, the role of miR-26a in distinct cancer cells depends on cellular context. Our research displayed the decreased expression of miR-26a in all of the 4 breast cancer cell lines as well as the clinical specimens of tumor. Meanwhile, the expression level of miR-26a was significantly correlated with tumor size, Her2 and Ki-27 status of breast cancer patients. In this study, to reveal the role of miR-26a in breast cancer cells *in vitro*, we tested the effects of miR-26a on cell growth. According to the results, miR-26a functioned as the tumor suppressor in breast cancer as it was shown to have the ability to inhibit breast cancer proliferation, colony formation, migration and induce cell apoptosis. In the sense, the role of miR-26a in breast cancer cells is similar to which in hepatocellular carcinoma [35], lung cancer [36], nasopharyngeal carcinoma [37], lymphoma [38], and contrary to which in T-cell lymphoblastic leukemia [39] and glioma [40].

To our knowledge, our study provides the first piece of evidence that miR-26a restoration reduced proliferation and induced apoptosis through repression of MCL-1. In various tumor types, especially in those miR-26a functions as a tumor suppressor, miR-26a was demonstrated to inhibit proliferation and colony formation through down-regulation of the histone-lysine N-methyltransferase, EZH2, a global regulator of gene expression. On the other hand, miR-26a was reported to facilitate carcinogenesis through the inhibition of PTEN. Among the predicted miR-26a targets in Targetscan, MCL-1 attracted our attention as it presented highly conservative base-pairing between the 3′-untranslated region of the mRNA in various species and the “seed sequence” located in the 5′ end of miR-26a, which is essential to determing whether targeting miRNA results in degradation of mRNA or inhibition of translation [41]. More importantly, MCL-1 is a pro-survival member of the Bcl-2 family, which has been studied intensively for the past decades owing to their importance in the regulation of apoptosis, tumorigenesis and cellular responses to anti-cancer therapy. Recently investigators determined that MCL-1 was targeted by several miRNAs in various types of cancer. [42], [43] Our study revealed that MCL-1 was overexpressed in breast cancer cells and ectopic restoration of miR-26a significantly decreased MCL-1 protein expression. In addition, miR-26a expression level was much higher in breast cancer tissues of MCL-1 negative group patients. Furthermore, functional studies by knockdown of MCL-1 phenocopied overexpression of miR-26a in breast cancer cells, resulting in cell growth and proliferation suppression *in vitro*, suggesting that the growth inhibitory effect of miR-26a was partly implemented by means of repressing MCL-1 expression.

Recently increasing miRNAs have been confirmed possessing potential tumor therapeutic functions [44] as they are involved in tumorigenesis and function as oncogenes or tumor suppressor genes. Therefore, strategies targeting miRNAs expression in cancer would be a new approach to cancer therapy. As we know paclitaxel is used in conventional chemotherapy to treat multiple cancers including breast cancer. Although individuals initially respond favourably, resistance can develop. Surmounting evidences have shown that treatment with paclitaxel alters the expression of genes that confer resistance, while these genes have been confirmed to be regulated by miRNAs [44]. Several research groups reported a novel therapeutic strategy to combine miRNAs gene therapy with chemotherapy. [45], [46] While the synergistic anti-tumor effects of several miRNAs with conventional chemotherapy have been reported, [47], [48] our research examined the synergistic anti-tumor effects of miR-26a with paclitaxel and the result suggested that miR-26a increased sensitivity to paclitaxel in breast cancer cells. Moreover, the study revealed that MCL-1 was involved in this function of miR-26a.

In conclusion, miR-26a was frequently down-regulated in breast cancer tissues and cell lines, which inhibited cell proliferation, colony formation and migration, but promoted apoptosis in breast cancer cells. These effects suggested miR-26a is a potential tumor suppressor in breast cancer. The investigation of the function mechanism indicated that miR-26a inhibited the tumor growth by at least partially targeting MCL-1. Furthermore, miR-26a effectively sensitized breast cancer cells to paclitaxel. The results of our exploration not only suggested the important role of miR-26a in breast cancer, but also indicated that therapeutic strategies aimed at restoration of miR-26a expression may be beneficial to patients with breast cancer.
