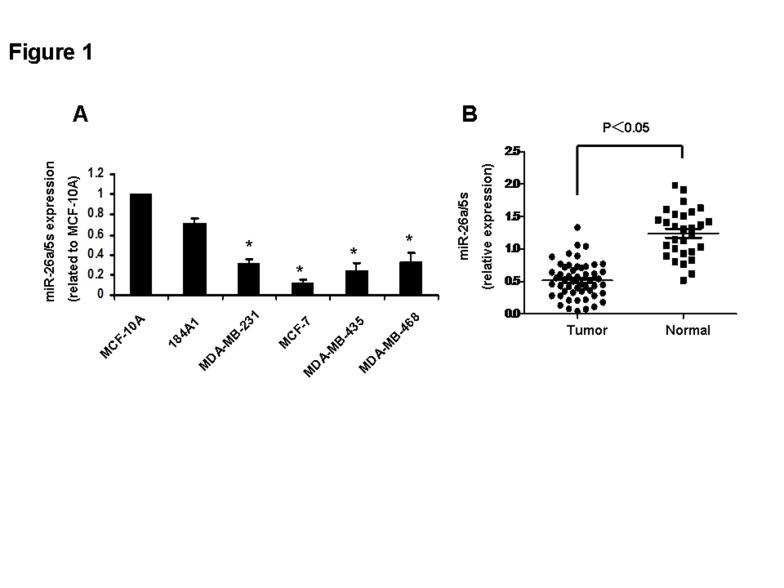# Correction: MiR-26a Inhibits Proliferation and Migration of Breast Cancer through Repression of MCL-1

**DOI:** 10.1371/annotation/4c8d2e73-67b1-473d-ae8e-4f847a5f7ef8

**Published:** 2013-12-20

**Authors:** Jie Gao, Laisheng Li, Minqing Wu, Min Liu, Xinhua Xie, Jiaoli Guo, Hailin Tang, Xiaoming Xie

In Figure 1, not all of the data is included. The incorrect version of the figure represents miR-26a expression analysis in 20 breast cancer specimens and 15 normal breast tissues. The revised version includes 52 breast cancer specimens and 29 normal breast tissues. Please see the revised version of Figure 1 here: 

**Figure:**